# Limited wedge resection for T1 colon cancer (LIMERIC-II trial) – rationale and study protocol of a prospective multicenter clinical trial

**DOI:** 10.1186/s12876-023-02854-9

**Published:** 2023-06-19

**Authors:** Julia Hanevelt, Jelle F. Huisman, Laura W. Leicher, Miangela M. Lacle, Milan C. Richir, Paul Didden, Joost M. J. Geesing, Niels Smakman, Jochim S. Terhaar Sive Droste, Frank ter Borg, A. Koen Talsma, Ruud W. M. Schrauwen, Bob J. van Wely, Ingrid Schot, Maarten Vermaas, Philip Bos, Colin Sietses, Wouter L. Hazen, Dareczka K. Wasowicz, David E. Ploeg, Dewkoemar Ramsoekh, Jurriaan B. Tuynman, Yasser A. Alderlieste, Rutger-Jan Renger, Ramon-Michel Schreuder, Johanne G. Bloemen, Ineke van Lijnschoten, Esther C. J. Consten, Daan J. Sikkenk, Matthijs P. Schwartz, Annelotte Vos, Jordy P. W. Burger, Bernhard W. M. Spanier, Nikki Knijn, Wouter H. de Vos Tot Nederveen Cappel, Leon M. G. Moons, Henderik L. van Westreenen

**Affiliations:** 1 Department of Gastroenterology and Hepatology, Isala, Dokter Van Heesweg 2, 28025 AB Zwolle, The Netherlands; 2 Department of Pathology, University Medical Center Utrecht, Utrecht, The Netherlands; 3 Department of Surgery, University Medical Center Utrecht, Utrecht, The Netherlands; 4 Department of Gastroenterology and Hepatology, University Medical Center Utrecht, Utrecht, The Netherlands; 5 Department of Gastroenterology & Hepatology, Diakonessenhuis Hospital, Utrecht, The Netherlands; 6 Department of Surgery, Diakonessenhuis Hospital, Utrecht, The Netherlands; 7 Department of Gastroenterology & Hepatology, Jeroen Bosch Ziekenhuis, Den Bosch, The Netherlands; 8 Department of Gastroenterology & Hepatology, Deventer Ziekenhuis, Deventer, The Netherlands; 9 Department of Surgery, Deventer Ziekenhuis, Deventer, The Netherlands; 10 Department of Gastroenterology & Hepatology, Ziekenhuis Bernhoven, Uden, The Netherlands; 11 Department of Surgery, Ziekenhuis Bernhoven, Uden, The Netherlands; 12 Department of Gastroenterology & Hepatology, IJsselland Ziekenhuis, Capelle a/d Ijssel, The Netherlands; 13 Department of Surgery, IJsselland Ziekenhuis, Capellle a/d Ijssel, The Netherlands; 14 Department of Gastroenterology & Hepatology, Ziekenhuis Gelderse Vallei, Ede, The Netherlands; 15 Department of Surgery, Ziekenhuis Gelderse Vallei, Ede, The Netherlands; 16 Department of Gastroenterology & Hepatology, Elisabeth-Tweesteden Ziekenhuis, Tilburg, The Netherlands; 17 Department of Surgery, Elisabeth-Tweesteden Ziekenhuis, Tilburg, The Netherlands; 18 Department of Pathology, Elisabeth-Tweesteden Ziekenhuis, Tilburg, The Netherlands; 19 Department of Gastroenterology & Hepatology, Amsterdam UMC Location VUmc, Amsterdam, The Netherlands; 20 Department of Surgery, Amsterdam UMC Location VUmc, Amsterdam, The Netherlands; 21 Department of Gastroenterology & Hepatology, Beatrixziekenhuis - Rivas, Gorinchem, The Netherlands; 22 Department of Surgery, Beatrixziekenhuis - Rivas, Gorinchem, The Netherlands; 23 Department of Gastroenterology & Hepatology, Catharina Ziekenhuis, Eindhoven, The Netherlands; 24 Department of Surgery, Catharina Ziekenhuis, Eindhoven, The Netherlands; 25 Eurofins/PAMM NL, Veldhoven, The Netherlands; 26 Department of Surgery, Meander Medisch Centrum, Amersfoort, The Netherlands; 27 Department of Gastroenterology & Hepatology, Meander Medisch Centrum, Amersfoort, The Netherlands; 28 Department of Pathology, Meander Medisch Centrum, Amersfoort, The Netherlands; 29 Department of Surgery, Rijnstate Hospital, Arnhem, The Netherlands; 30 Department of Gastroenterology & Hepatology, Rijnstate Hospital, Arnhem, The Netherlands; 31 Pathology DNA, Location Arnhem, The Netherlands; 32 Department of Surgery, Isala, Zwolle, The Netherlands

**Keywords:** T1 colorectal cancer, Early-stage colorectal cancer, Combined endo-laparoscopic surgery, Colonoscopy-assisted laparoscopic wedge resection

## Abstract

**Background:**

The sole presence of deep submucosal invasion is shown to be associated with a limited risk of lymph node metastasis. This justifies a local excision of suspected deep submucosal invasive colon carcinomas (T1 CCs) as a first step treatment strategy. Recently Colonoscopy-Assisted Laparoscopic Wedge Resection (CAL-WR) has been shown to be able to resect pT1 CRCs with a high R0 resection rate, but the long term outcomes are lacking. The aim of this study is to evaluate the safety, effectiveness and long-term oncological outcomes of CAL-WR as primary treatment for patients with suspected superficial and also deeply-invasive T1 CCs.

**Methods:**

In this prospective multicenter clinical trial, patients with a macroscopic and/or histologically suspected T1 CCs will receive CAL-WR as primary treatment in order to prevent unnecessary major surgery for low-risk T1 CCs. To make a CAL-WR technically feasible, the tumor may not include > 50% of the circumference and has to be localized at least 25 cm proximal from the anus. Also, there should be sufficient distance to the ileocecal valve to place a linear stapler. Before inclusion, all eligible patients will be assessed by an expert panel to confirm suspicion of T1 CC, estimate invasion depth and subsequent advise which local resection techniques are possible for removal of the lesion. The primary outcome of this study is the proportion of patients with pT1 CC that is curatively treated with CAL-WR only and in whom thus organ-preservation could be achieved. Secondary outcomes are 1) CAL-WR’s technical success and R0 resection rate for T1 CC, 2) procedure-related morbidity and mortality, 3) 5-year overall and disease free survival, 4) 3-year metastasis free survival, 5) procedure-related costs and 6) impact on quality of life. A sample size of 143 patients was calculated.

**Discussion:**

CAL-WR is a full-thickness local resection technique that could also be effective in removing pT1 colon cancer. With the lack of current endoscopic local resection techniques for > 15 mm pT1 CCs with deep submucosal invasion, CAL-WR could fill the gap between endoscopy and major oncologic surgery. The present study is the first to provide insight in the long-term oncological outcomes of CAL-WR.

**Trial registration:**

CCMO register (ToetsingOnline), NL81497.075.22, protocol version 2.3 (October 2022).

## Background

A segmental colon resection with lymph node dissection is associated with significant morbidity (24%) and mortality (2%) [1], regardless of T-stage. In T1 colorectal cancer (CRC), three independent histological features have been identified to be risk factors for lymph node metastases (LNM), i.e. (lympho-) vascular invasion, poor (high-grade) differentiation (G3) and high-grade tumor budding (Bd2-Bd3) [2–7]. A completely removed (R0) T1 CRC can be classified as low risk T1 when histological examination shows none of the high risk factors for LNM. Prior retrospective studies report a very low risk of LNM (1%) for low risk T1 CRC [8–11]. As this negligible risk does not outweigh the significant morbidity and mortality of an segmental resection, patients with low risk T1 CRC are sufficiently treated with a local resection.

As upfront risk stratification is not possible, all patients with suspected T1 CRC could receive initial local treatment according to current national guidelines. An additional segmental resection with lymph node dissection is only indicated if histological examination shows high risk factors for LNM or when an incomplete local resection (R1) is performed. It has been proven that secondary surgery after initial local endoscopic resection (or an attempt) has no negative effects on the development of LNM or cancer recurrence [8, 12–16]. Therefore a primary local en-bloc resection strategy in order to prevent unnecessary upfront major surgery for low risk T1 CC patients is justified. Moreover, patients who underwent local endoscopic therapy have similar quality of life (QoL) without increased fear of cancer recurrence compared to patients who underwent a segmental resection [17].

In contrast to previous insights, the presence of deep invasion is no longer recognized as high risk factor for LNM. A suspected deeply-invasive T1 colon carcinoma (CC) is therefore not a strong indicator for a segmental resection [18]. Unfortunately, the therapeutic options to perform a ‘’full thickness’’ local resection in the colon are scarce. The R0-resection rate and safety of an endoscopic submucosal dissection (ESD) in the colon is significantly compromised in case of deeply invasive lesions and therefore not recommended in these cases [19, 20]. This means that the endoscopic Full Thickness Resection (eFTR) is currently the only available technique to remove deeply-invasive T1 colon carcinomas (CC) up to 15 mm [21].

Recently, we have shown that Colonoscopy-Assisted Laparoscopic Wedge Resection (CAL-WR) is an effective and safe technique to remove endoscopically unresectable benign colonic lesions (LIMERIC-trial [22]). The technical success rate of CAL-WR was 93% and a R0-resection was achieved in 91% of the patients with low morbidity (6%) and no mortality. Subsequently, CAL-WR was introduced as a feasible ‘’full-thickness’’ resection technique for the removal of suspected T1 CCs [23], however long-term oncological outcomes of CAL-WR are yet unclear. In particular for large deep-invasive lesions, CAL-WR could fill the gap between the current endoscopic resection techniques and major oncologic surgery, providing more patients an minimal invasive organ-preserving treatment option.

The aim of this study is to prospectively register the effectiveness, safety, and costs of CAL-WR as primary treatment of suspected (deep-invasive) T1 CC and to provide insight in its long-term oncological outcomes.

## Methods

In this prospective multicenter clinical trial, patients with suspected T1 CC (after assessment by an expert panel) will receive CAL-WR as initial treatment in order to prevent unnecessary segmental resections for low risk T1 CC.

### Study population

*In order to be eligible for inclusion, patients must meet the following inclusion criteria:*

- Non-pedunculated lesion which is macroscopically suspected for (deep-invasive) T1 colon carcinoma during endoscopy (Hiroshima C1-3 [24]) and/or histologically proven adenocarcinoma
- Size < 40 mm (endoscopic assessment)
- Localized at least > 25 cm proximal from the anus (measured endoscopically)
- Localized with sufficient distance to the ileocecal valve to place the linear stapler
-  > 18 years old

*A potential subject who meets any of the following criteria will be excluded from participation in this study:*

- Rectal carcinoma
- Distant metastasis at baseline
-  > 50% circumferential growth of the lesion
- Prior local endoscopic resection or attempt (lifting not included)
- History of malignancy in the past 5 years or current presence of another malignancy

### Objectives

The primary objective of this study is to determine the proportion of patients with pT1 CC for whom CAL-WR was shown to be formerly a curative treatment (i.e. low-risk T1 CC, R0) and in whom an (additional) segmental resection could thus be prevented. Secondary objectives are to evaluate CAL-WR’s technical success and R0 resection rate for T1 CC, procedure-related morbidity and mortality, the 5-year overall and disease free survival, 3-year metastases free survival, its costs, and the impact on QoL.

### Study design

This study is a prospective multicenter clinical trial in The Netherlands. Patients with a macroscopic suspected and/or histological suspected T1 CC, meeting the in-and exclusion criteria, are eligible for inclusion. Based upon endoscopic images, the expert panel decide whether a lesion is suspect for a T1 CC. To reduce subjectivity and standardize the assessment, all expert panel members will use internationally applied scoring systems; Paris classification [25], Hiroshima classification [24] and report the morphological malignant features that are present. In particular the presence of a demarcated area with neoplastic pit pattern (Hiroshima C1-3) will weigh heavy when a lesion is screened for eligibility by the expert panel [26]. Depending on the macroscopic assessment and estimated depth of invasion, the expert panel will state if the lesion is suspected for T1 CC (superficial or deeply-invasive) and subsequently recommend which local resection strategie(s) (ESD, eFTR or CAL-WR) is/are suitable.

**Fig. 1:**
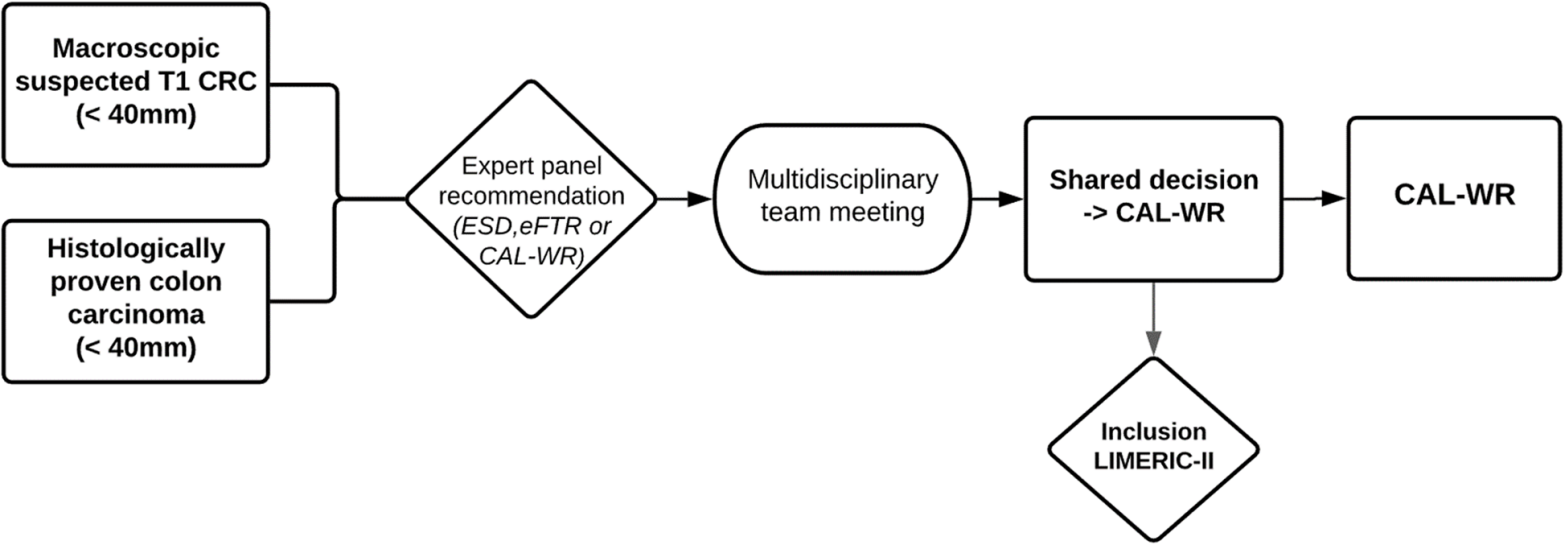
Flowchart patient enrollment. Legend: ESD—Endoscopic submucosal dissection, eFTR—Endoscopic full-thickness resection, CAL-WR—Colonoscopic-Assisted Laparoscopic Wedge Resection

According to the recommendation of the current national Dutch colorectal carcinoma guideline, all suspected T1 CCs must be discussed in the local multidisciplinary team meeting to determine the best treatment strategy. If CAL-WR is regarded to be a suitable treatment by all parties, technically possible and preferred by the patient, inclusion of the patient will follow (Fig. 1). All eligible patients rejected for inclusion by the expert panel will be registered in a screening-log. Preoperative measurement of CEA (carcinoembryonic antigen) is required. In accordance with the current Dutch T1 CRC guideline, tumor staging through imaging of the chest and abdomen is not recommended in case of suspected T1 CRCs and therefore not indicated before the procedure. Written informed consent will be obtained and the patient and procedure related items will be registered in a web-based database (ResearchManager, Version 6.5). The estimated duration of inclusion will be 24 months and the total follow-up period of each patient is 5 year.

### Quality assurance

#### Expert panel

Patients with an macroscopic and/or histologically suspected T1 colon carcinoma, meeting the inclusion criteria, will be submitted to a multidisciplinary expert panel to evaluate each case for eligibility. This panel will consist of two surgeons and five gastroenterologists. The gastroenterologists, who are specialized in endoscopic removal of tumors by ESD/eFTR and work in different participating hospitals, will assess the chance of malignancy using international standardized scoring systems [24, 25, 27]. Subsequently, all experts report which local resection methods are possible in the particular case (eFTR/ESD/CAL-WR). Inclusion is only possible when at least three gastroenterologists of the expert panel conclude that the lesion is suspected for T1 CC and eligible for removal through CAL-WR, and when one of the surgeons considers a CAL-WR technically feasible. For thorough evaluation, endoscopic images of the tumor will be sent including overview and close up images and with use of advanced imaging techniques such as narrow band imaging. To minimalize inclusion of T2 colon carcinomas, all gastroenterologist in the expert panel will be trained to distinguish deep invasive submucosal (T1) from muscularis propria (T2) colorectal cancer, by use of the recently developed scoring system by Koyama et al. [28]. All individual reports of the expert panel members will be archived in the study database. (Paris classification, Hiroshima classification, morphological malignant features that are present, morphological features suspected for T2 CC, their individual conclusion (benign, suspected T1 CC or suspected for ≥ T2) and recommendation for treatment).

#### Participating surgeons

All participating surgeons must be experienced in laparoscopic colorectal surgery. Aiming to further standardize the procedure, all participating surgeons must have attended an e-learning concerning the technique and the possible pitfalls of the technique. However, the majority of the surgeons is already familiar with CAL-WR by participating in our previous trial [22]. Patients will be informed by the surgeon about the possibility of conversion into an laparoscopic or open major oncologic resection if perioperative CAL-WR is not feasible.

### CAL-WR technique

**Fig. 2:**
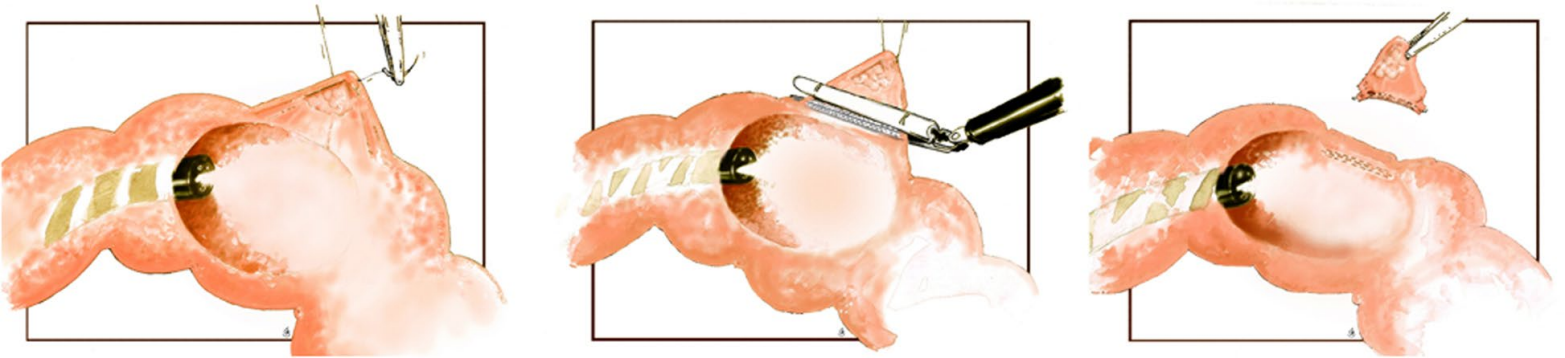
Colonoscopic-Assisted Laparoscopic Wedge Resection (CAL-WR) procedure

CAL-WR is a minimally invasive technique to locally resect lesions by using a linear stapler without making an anastomosis (Fig. 2). All included patients will undergo split-dose bowel preparation before the procedure. Patients are placed in French position under general anesthesia. The procedure is initiated with a diagnostic laparoscopy with the insertion of three trocars. At first, the spot in the colon is identified and the corresponding part of the colon will be mobilized. This is to ensure the ability to place the linear stapler, to make a CAL-WR possible. Secondly, the colonoscopy is performed. A suture is laparoscopically placed near the tumor with intraluminal endoscopic visualization. In case of a tumor close to the mesentery, the colonic wall can be dissected from the mesentery with preservation of the marginal artery. Traction is provided on the suture to enable positioning of the linear stapler. Before stapling off the tumor, patency of the colonic lumen (or the lumen of the ileum in case of a cecal lesion) as well as the total inclusion of the tumor is confirmed endoscopically. The resected specimen is then removed in an endobag through the 12 mm trocart. Lastly, the peritoneal surface as well as the luminal aspect of the colon is inspected for signs of bleeding or perforation before ending the procedure. Total operation time, used materials, and duration of endoscopy are reported in the online case report form.

### Histopathological examination

Over the last years, a nation-wide cohort of pathologists with special interest in gastro-intestinal oncology were extensively trained to score the above and below mentioned histological risk factors, in the context of the PATCH-study (https://t1crc.com/research—KWF Dutch Cancer Society reference number: 12723 / 2019–2). To guarantee a high-quality histopathological assessment, all the resected specimen will be judged by PATCH-trained dedicated pathologist(s) of the participating centers.

The specimen will be processed through the following instructions (standard care). The pathologist receives the fresh resected specimen in toto, without manipulation of the staple line by the surgeon. The pathologist removes the staples, the lateral margins and deep peritoneal aspect will be inked with different colors. Thereafter, the specimen will be stretched on a paraffin block (or mesh), photographed, and fixed for 24 h at room temperature. After fixation, longitudinal sections of limited length and width of the whole specimen will be included and the specimen will be accessioned in toto. Histological diagnosis of the tumor is carried out in accordance with the current guidelines and will include histological type, invasion depth, tumor grade, presence of vascular/lymph invasion, grade of budding and margin status. Incomplete resection is defined as a resection margin of less than 0.1 mm (R1) [29] and/or when radicality cannot be determined (Rx). T1 CCs with the presence of one or more histological risk factors, i.e. (lympho-) vascular invasion, poor (high-grade) differentiation (G3) and high-grade tumor budding (Bd2-Bd3), are classified as high-risk T1 CC.

### Follow-up

**Fig. 3:**
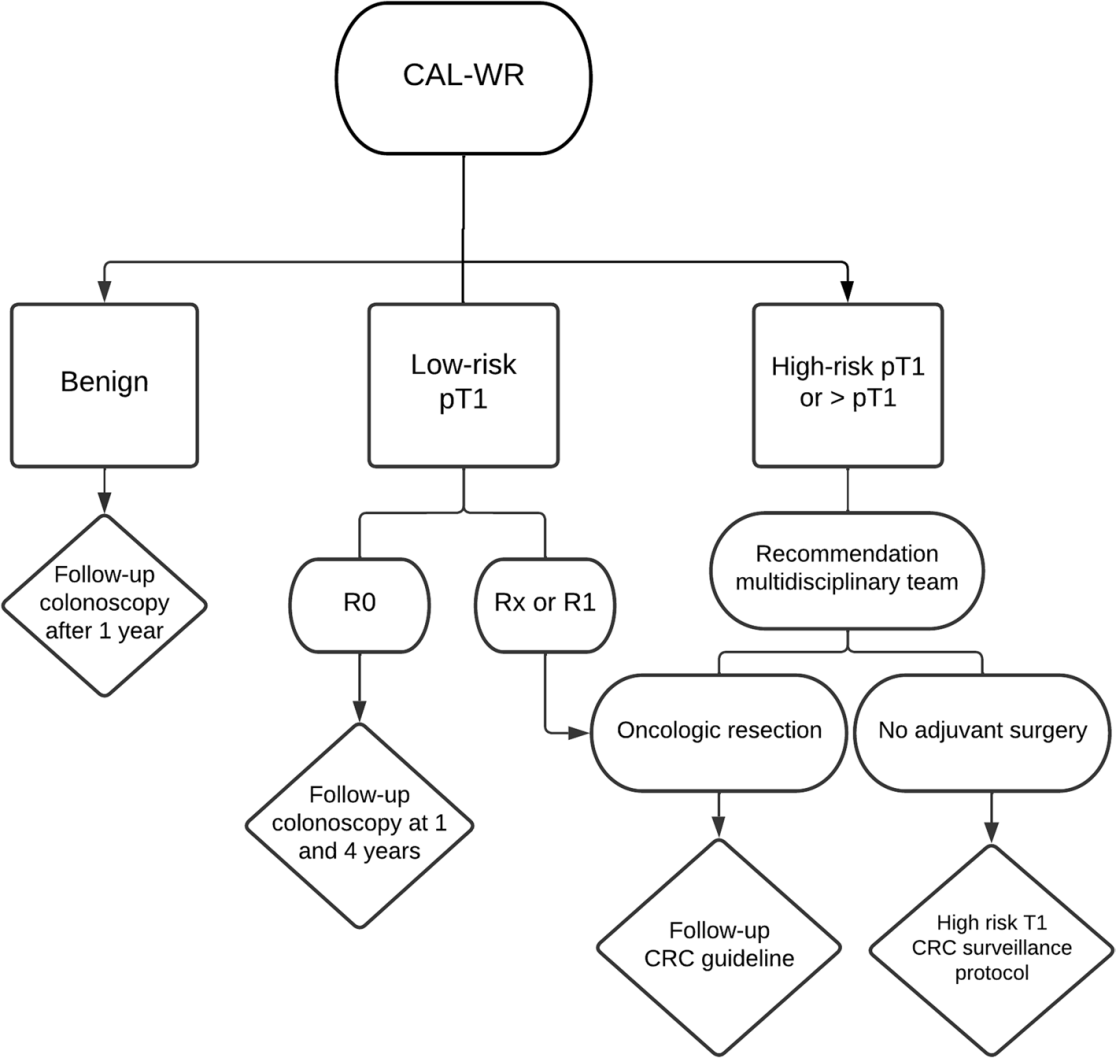
Flowchart follow-up. Legend: CAL-WR—Colonoscopic-Assisted Laparoscopic Wedge Resection, Low risk pT1 – pT1 colon carcinoma without (lympho-) vascular invasion, poor (high-grade) differentiation and high-grade tumor budding, R0 – radical resection, R1 – irradical resection (resection margin of less than 0.1 mm), Rx—radicality could not be determined, High risk pT1—pT1 colon carcinoma with one or more of the following histological risk factors: (lympho-) vascular invasion, poor (high-grade) differentiation and high-grade tumor budding, CRC – colorectal cancer

After CAL-WR and subsequent histological examination, 3 groups will be formed i.e. (1) benign pathology, (2) low risk T1 CC (i.e. carcinomas with absence of histological high risk features for lymph node metastasis), and (3) high risk T1 CC (i.e. carcinomas with histological high risk features for lymph node metastasis) or accidentally found ≥ pT2 (Fig. 3). All patients diagnosed with a benign colonic polyp, will receive a follow-up colonoscopy after 1 year to evaluate local recurrence rate. Patients diagnosed with low risk T1 CC will undergo colonoscopy 12 and 48 months after curative radical (R0) resection, according to the current national guideline. In case patients are diagnosed with an incomplete resected low risk 1 CC, high risk T1 CC or ≥ pT2, completion surgery is advised. All patients with an indication for completion surgery will be staged with chest and abdomen imaging by computer tomography (CT) to rule out potential synchronous metastasis and will subsequently be discussed in the multidisciplinary team meeting. If completion surgery is considered to be beneficial, it needs to be performed within six weeks after initial CAL-WR in order to guarantee the patients’ oncological outcome [30]. In case of high complication risks and/or based on the patients’ choice, there can be refrained from a complementary resection and give preference to intensive follow-up. The impact of CAL-WR on QoL will be evaluated in all patients, also those that undergo completion surgery, using three questionnaires (EQ-5D-5L, QLQ CR29 and CWS). The questionnaires will be performed at baseline, at 3 and 36 months after the procedure. Participation in the national Prospectief Landelijk CRC cohort (PLCRC) is preferable for the questionnaires [31].

### Outcomes

#### Primary endpoint

Proportion of patients with pT1 colon carcinoma for whom CAL-WR was shown to be a curative treatment (i.e. low-risk T1 CC, R0) and in whom an (additional) segmental resection could thus be prevented (percentage of patients).

#### Secondary endpoints

1. Effectiveness (technical success and macroscopic- and microscopic radical resection)
2. 30-day postoperative morbidity and mortality rate. Complications will be assessed according to the Clavien-Dindo classification. Clavien-Dindo grade I and II complications will be classified as minor and grade III-V complications as major.
3. 3-year metastasis free survival defined as the development of metastasis outside the area of the intended surgery (intensive surveillance), or after completion surgery.
4. 5-year overall survival and disease free survival (disease free survival is defined as the time between surgery until local cancer recurrence or detection of distant metastasis)
5. Total procedure related and postoperative costs (‘in-hospital’ costs between the primary day of surgery until 30-days after discharge)
6. Postoperative quality of life

### Sample size calculation

The sample size was determined based on a (one-proportion) power calculation assuming a proportion of 45% of all patients with pT1 CC to be cured with local resection (proportion low-risk T1 CC, R0 resection). For the sample size calculation, we used a desired width of a two-sided 95% confidence interval of 0.2. This calculation resulted in a sample size of 96 patients with pT1 CC. As we estimate to exclude 15% of the patients because of a pT2 CC, 10% due to benign histology and an overall technical failure in 10% [22], a total sample size of 143 patients is calculated. We will perform a mid-term evaluation at half the target number of inclusions. If the proportion of patients with pT1 CC is lower than 60% at that stage, the inclusion will be temporarily suspended and the principal investigators will critically assess the current mode of inclusion.

### Data analysis

Data on demographic and baseline characteristics will be summarized for continuous variables, in case of normal distribution by mean and standard deviation (SD), and in case of non-normal distribution by median and (interquartile) range. Discrete variables and categorical data will be presented as proportions of patients (percentages). All baseline characteristics and differences in secondary outcomes, such as morbidity and postoperative QoL, will be compared between patients that only received CAL-WR versus patients that received also additional oncologic resection, using the Student’s T-test (in case of normally-distributed continuous data) or the Mann–Whitney U test (in case of non-parametrical continuous data). Normality will be verified using the Kolmogorov–Smirnov test. A (two-sided) *p*-value < 0.05 will be considered significant. The primary endpoint will be described as the proportion (percentage with 95% confidence interval) of patients with pT1 CC where only local resection with CAL-WR appeared to be curative and thereby oncologic resection was prevented. The 5-year overall survival and disease free survival will be assessed using a Kaplan–Meier analysis*.* The 30-day postoperative morbidity and mortality will described as proportions (percentages). Data analysis will be performed by using the Statistical Program for the Social Sciences (SPSS) version 28.0 (IBM. Corp, Armonk, New York, USA).

The cost analysis will be conducted from a hospital’s perspective. Total procedure-related cost of CAL-WR will be calculated in Euro. Healthcare costs will be calculated by multiplying used healthcare services by the appropriated unit cost prices. Bottom-up micro costing will be applied. For all other healthcare services reference prices, will be used where available. Costs will be discounted to correct for inflation. Eventually, the cost efficiency of our strategy will be calculated, considering also the number of patients that will need two operations i.e. patients with high risk T1 or ≥ T2.

### Ethics

The study protocol is approved by the Medical Ethics Committee of the Isala clinics, Zwolle (NL81497.075.22). The study will be conducted according to the rules on medical research involving human subjects (Medical Research (Human Subjects) Act), in Dutch: Wet Medisch Wetenschappelijk Onderzoek met mensen (WMO) and the principles of the Declaration of Helsinki (64th WMA General Assembly, Fortaleza, October 2013).

## Discussion

The substantially rising incidence of stage I CRC emphasizes the urgent need for a more widely applicable local resection technique in order to prevent unnecessary major oncologic surgery. This prospective multicenter study aims to give insight in the effectiveness, safety, and costs of CAL-WR as local resection technique for T1C CCs. Furthermore, patients will be followed for a total duration of five years, allowing us to evaluate the short and long-term oncological outcome of CAL-WR as primary resection strategy.

The biggest challenge in this study is to include patients with T1 CC as accurately as possible. Identification of malignant morphologic features such as depression, excavation and spontaneous bleeding, combined with evaluation of the vascular pattern using the Hiroshima Classification, is currently used in standard clinical practice to differentiate between adenomas and T1 CCs. In a large multicenter study in the Netherlands, Backes et al. achieved a sensitivity of 78.7% with a specificity of 94.2% using this standardized assessment [27]. Optical discrimination between deep submucosal invasive T1 and T2 CC is also challenging. To minimize the inclusion of benign or ≥ T2 lesions, we implemented an expert panel of five dedicated gastroenterologist to reassess all eligible patients. In order to reduce inter-observer variation, each independent member of the expert panel, as well as the referring gastroenterologist, will estimate the chance of T1 malignancy using international scoring systems with low-threshold use of the risk stratification developed by Backes et al. [27]. Moreover, our expert panel will be trained to macroscopically differentiate between deep submucosal invasive CCs and ≥ T2 CCs by using a scoring system composed of five endoscopic findings which was developed by Koyama et al. [28]. These 5 endoscopic findings (deep depression, demarcated depressed area, fold convergency, erosion or white plaque, and Borrmann type 2 or 3 tumor) combined in a scoring system resulted in a sensitivity and specificity of respectively 82% and 83% in their development cohort. Despite the precautions taken in this study protocol to limit inclusion of adenomas and ≥ T2 colon carcinomas, it shall be unavoidable to include purely T1 CCs. This margin is taken into account in our sample size calculation.

In contrast to earlier insights, deep submucosal invasion (T1b) is not considered as independent risk factor for lymph node metastasis [18]. In the absence of other histological risk features (i.e. poorly differentiation, high grade tumor budding, (lympho-)vascular invasion), patients with deep submucosal invasive T1 colon carcinomas have no longer an indication for major oncologic surgery. Therefore, deep submucosal invasion was not included as criterion to advice an additional oncologic resection after radical CAL-WR in this protocol. Prior endoscopic resection or attempt is in this study handled as exclusion criterion to guarantee an unbiased sample population. However, outside study context CAL-WR is also suited to perform scar excisions of irradical endoscopically removed low-risk pT1 colon carcinoma. In the recent prospective clinical trial by Leicher et al. [22, 32], a technical success rate of 90% was achieved with R0 resection rates variating from 86%-92% for irradical resected low-risk T1 colon carcinomas, however numbers were small (*n* = 24).

In the study of Leicher et al., macroscopic recurrent tissue was found during follow up colonoscopy in four cases of which three cases were initial radical (R0) resections. The indication to perform CAL-WR in these patients were either a difficult location of the polyp or a non-lifting polyp. In all these patients, initial and recurrence histology was benign. Considering this recurrence rate, all patients with definite benign histology in this study, will receive a follow-up colonoscopy 1 year after CAL-WR, regardless of current surveillance guidelines.

A significant part of patients with suspected T1 CC are not eligible for removal by eFTR due to the size of the lesion. Expertise with ESD in the proximal colon is often not available in non-academic hospitals. Moreover, ESD leads more often to an incomplete resection in case of deep submucosal invasion [19]. Our hypotheses is that CAL-WR will safely expand the local therapeutic armamentarium that is currently available for T1 colon carcinomas. In particular for deep-invasive CCs, CAL-WR could fill in the gap between endoscopic minimally invasive local resection techniques and major oncologic surgery, preventing unnecessary major oncologic surgery.

## Data Availability

The datasets generated and/or analyzed during this study are not publicly available due to individual privacy but are available from the corresponding author on reasonable request.

## Abbreviations

CAL-WR: Colonoscopy-Assisted Laparoscopic Wedge Resection
CC: Colon carcinoma
CEA: Carcinombryonic antigen
CRC: Colorectal cancer
eFTR: Endoscopic Full Thickness Resection
ESD: Endoscopic Submucosal Dissection
LNM: Lymph-node metastases
PLCRC: Prospective Dutch Colorectal Cancer (PLCRC) cohort
QoL: Quality of Life
