# Correction: Limited wedge resection for T1 colon cancer (LIMERIC-II trial) – rationale and study protocol of a prospective multicenter clinical trial

**DOI:** 10.1186/s12876-023-02905-1

**Published:** 2023-07-27

**Authors:** Julia Hanevelt, Jelle F. Huisman, Laura W. Leicher, Miangela M. Lacle, Milan C. Richir, Paul Didden, Joost M. J. Geesing, Niels Smakman, Jochim S. Terhaar Sive Droste, Frank ter Borg, A. Koen Talsma, Ruud W. M. Schrauwen, Bob J. van Wely, Ingrid Schot, Maarten Vermaas, Philip Bos, Colin Sietses, Wouter L. Hazen, Dareczka K. Wasowicz, David E. Ploeg, Dewkoemar Ramsoekh, Jurriaan B. Tuynman, Yasser A. Alderlieste, Rutger-Jan Renger, Ramon-Michel Schreuder, Johanne G. Bloemen, Ineke van Lijnschoten, Esther C. J. Consten, Daan J. Sikkenk, Matthijs P. Schwartz, Annelotte Vos, Jordy P. W. Burger, Bernhard W. M. Spanier, Nikki Knijn, Wouter H. de Vos Tot Nederveen Cappel, Leon M. G. Moons, Henderik L. van Westreenen

**Affiliations:** 1 Department of Gastroenterology and Hepatology, Dokter Van Heesweg 2, Isala, Zwolle, 28025 AB The Netherlands; 2 Department of Pathology, University Medical Center Utrecht, Utrecht, The Netherlands; 3 Department of Surgery, University Medical Center Utrecht, Utrecht, The Netherlands; 4 Department of Gastroenterology and Hepatology, University Medical Center Utrecht, Utrecht, The Netherlands; 5 Department of Gastroenterology & Hepatology, Diakonessenhuis Hospital, Utrecht, The Netherlands; 6 Department of Surgery, Diakonessenhuis Hospital, Utrecht, The Netherlands; 7 Department of Gastro- enterology & Hepatology, Jeroen Bosch Ziekenhuis, Den Bosch, The Netherlands; 8 Department of Gastroenterology & Hepatology, Deventer Ziekenhuis, Deventer, The Netherlands; 9 Department of Surgery, Deventer Ziekenhuis, Deventer, The Netherlands; 10 Department of Gastroenterology & Hepatology, Ziekenhuis Bernhoven, Uden, The Netherlands; 11 Department of Surgery, Ziekenhuis Bernhoven, Uden, The Netherlands; 12 Department of Gastroenter ology & Hepatology, IJsselland Ziekenhuis, Capelle a/d Ijssel, The Netherlands; 13 Department of Surgery, IJsselland Ziekenhuis, Capellle a/d Ijssel, The Netherlands; 14 Department of Gastroenterology & Hepatology, Ziekenhuis Gelderse Vallei, Ede, The Netherlands; 15 Department of Surgery, Ziekenhuis Gelderse Vallei, Ede, The Netherlands; 16 Department of Gastroenterology & Hepatology, Elisabeth-Tweesteden Ziekenhuis, Tilburg, The Netherlands; 17 Department of Surgery, Elisabeth-Tweesteden Ziekenhuis, Tilburg, The Netherlands; 18 Department of Pathology, Elisabeth-Tweesteden Ziekenhuis, Tilburg, The Netherlands; 19 Department of Gastroenterology & Hepatology, Amsterdam UMC Location VUmc, Amsterdam, The Netherlands; 20 Department of Surgery, Amsterdam UMC Location VUmc, Amsterdam, The Netherlands; 21 Department of Gastroenterology & Hepatology, Beatrixziekenhuis - Rivas, Gorinchem, The Netherlands; 22 Department of Surgery, Beatrixziekenhuis - Rivas, Gorinchem, The Netherlands; 23 Department of Gastroenterology & Hepatology, Catharina Ziekenhuis, Eindhoven, The Netherlands; 24 Department of Surgery, Catharina Ziekenhuis, Eindhoven, The Netherlands; 25 Eurofns/PAMM NL, Veldhoven, The Netherlands; 26 Department of Surgery, Meander Medisch Centrum, Amersfoort, The Netherlands; 27 Department of Gastroenterology & Hepatology, Meander Medisch Centrum, Amersfoort, The Netherlands; 28 Department of Pathology, Meander Medisch Centrum, Amersfoort, The Netherlands; 29 Department of Surgery, Rijnstate Hospital, Arnhem, The Netherlands; 30 Department of Gastroenterology & Hepatology, Rijnstate Hospital, Arnhem, The Netherlands; 31 Pathology DNA, Location Arnhem, The Netherlands; 32 Department of Surgery, Isala, Zwolle, The Netherlands

**Correction:** ***BMC Gastroenterol*** **23, 214 (2023).**

10.1186/s12876-023-02854-9.

Following publication of the original article [1], it was reported that David E. Ploeg’s name was incorrectly published as David E. van der Ploeg.

The correct authorship list is given in this Correction article and the original article has been updated.
